# LiDAR-Based Intensity-Aware Outdoor 3D Object Detection

**DOI:** 10.3390/s24092942

**Published:** 2024-05-06

**Authors:** Ammar Yasir Naich, Jesús Requena Carrión

**Affiliations:** School of Electronic Engineering and Computer Science, Queen Mary University of London, London E1 4NS, UK

**Keywords:** 3D object detection, deep learning, lidar intensity, computer vision, LiDAR

## Abstract

LiDAR-based 3D object detection and localization are crucial components of autonomous navigation systems, including autonomous vehicles and mobile robots. Most existing LiDAR-based 3D object detection and localization approaches primarily use geometric or structural feature abstractions from LiDAR point clouds. However, these approaches can be susceptible to environmental noise due to adverse weather conditions or the presence of highly scattering media. In this work, we propose an intensity-aware voxel encoder for robust 3D object detection. The proposed voxel encoder generates an intensity histogram that describes the distribution of point intensities within a voxel and is used to enhance the voxel feature set. We integrate this intensity-aware encoder into an efficient single-stage voxel-based detector for 3D object detection. Experimental results obtained using the KITTI dataset show that our method achieves comparable results with respect to the state-of-the-art method for car objects in 3D detection and from a bird’s-eye view and superior results for pedestrian and cyclic objects. Furthermore, our model can achieve a detection rate of 40.7 FPS during inference time, which is higher than that of the state-of-the-art methods and incurs a lower computational cost.

## 1. Introduction

The pace of research into 3D vision perception has accelerated over the past few years, as it is an essential component of indoor and outdoor navigation systems. Examples of applications of navigation systems include autonomous vehicles (AVs) [1,2,3], robots [4,5], and augmented reality [6]. In regard to AVs, 3D perception in outdoor, urban environments still remains an open challenge [1,7,8]. This challenge is even greater in very complex scenarios, such as in dense intersections with a high volume of traffic and uncertain pedestrian actions. To develop AVs that operate safely and in a hazard-free manner, it is important to understand what AVs perceive in environments that present on-road and off-road traffic objects, especially in dense and occluded environments. In AVs, 3D visual perception LiDAR and stereo camera sensors are considered the primary choices of sensing modalities. Unlike 2D stereo camera images, LiDAR 3D point clouds provide accurate depth information about the surrounding objects, such as object scale, relative positions, and occlusion. However, due to the inherent sparsity and higher density variance in 3D point cloud data, it is very difficult to capture the geometric abstraction of objects. To this end, different point-cloud-encoding techniques have been proposed that implement sparse-to-dense feature representation conversion while preserving geometric abstraction. The proposed encoders are then followed by 2D convolution filters for object detection and localization [9,10,11,12].

Recent 3D detection and localization methods rely on geometric encoding techniques for feature extraction [2,10,11,13,14]. Geometric encoding consumes all the points in a 3D cloud or a subset of points resulting from quantization. The existence of false and noisy points in a 3D cloud can have a detrimental impact on the performance of geometric encoding techniques for feature extraction [15,16]. Specifically, 3D point clouds from LiDAR are highly susceptible to scattering media such as fog, snow, rain, or dust in outdoor environments [17,18,19,20,21], and in these scenarios, feature extraction becomes more challenging. To improve feature extraction in noisy point clouds, denoising methods have been developed [18,19,22]. However, these methods are computationally expensive, and their efficiency has not been evaluated on standard benchmark datasets [7,23,24,25].

To develop robust feature extraction methods for 3D object detection, we argue that voxel-wise point cloud intensity histograms can complement geometric features, as false and true points might have different intensity properties. In this paper, we propose an intensity-based encoder for extracting voxel-wise features, which are then used in a 3D and 2D backbone detection network. Our intensity-based encoder integrates the underlying geometric structure with the intensity histogram to produce sparse feature maps. Using a custom Cuda kernel, we generate voxel-wise intensity histograms in a parallel fashion to increase efficiency. The extracted sparse feature map is followed by a sparse convolution stage that produces a dense feature map. Finally, a Region Proposal Network (RPN) is used for classification and 3D bounding box estimation.

## 2. Related Work

Geometric feature representation from 3D point clouds can be broadly categorised into three point-cloud-encoding schemes, namely, grid-based, point-based, and hybrid. In this section, we review each of these categories.

### 2.1. Grid-Based Feature Encoders

Grid-based approaches apply fixed grid structures to 3D world scenes. This allows for point vectorization techniques to be subsequently used on a grid-cell basis. Grid-based encoding offers a trade-off between efficiency and memory size but incurs information loss due to the quantization of the grid size. Early attempts to encode irregular and sparse 3D point clouds imposed a regular structure onto the 3D space and performed sparse-to-dense feature conversion. Sparse-to-dense feature conversion was then followed by 2D convolutional neural networks (CNNs). In [26], 3D point clouds were projected onto a 2D bird’s-eye-view (BEV) plane to form a range image, after which a fully connected CNN was used to perform vehicle detection. Similarly, Refs. [27,28,29] used deep multi-sensor fusion for 3D detection and 2D BEV, resulting in more-robust schemes. VoxelNet [12] uses end-to-end 3D object detection on point clouds while using simple sampling techniques to suppress sparse regions and develop dense feature representations for 3D object detection. This approach was extended in [30] by fusing RGB and point clouds. Based on VoxelNet, the SECOND approach [31] improved the inference speed of VoxelNet at the expense of an increased complexity and memory footprint while using sparse 3D convolutions.

### 2.2. Point-Set-Based Feature Encoders

Unlike grid-based methods, point-set-based encoders consume all raw points while preserving the permutation invariance of the input point cloud. The authors of [10,11] used point-set-based techniques in combination with deep learning pipelines. PointRCNN [32] generates 3D bounding boxes by consuming raw point clouds using segmentation in the first stage of the model followed by 3D box refinement instead of using prior-fixed anchor boxes. In a related work [33], sparse-to-dense 3D object detection was used, reaching an improved detection rate of up to 10 frames per second (FPS). The EdgeConv model, proposed in [34], used a dynamic graph-based technique on raw point clouds and achieved better discrimination of local features than that demonstrated in [10]. However, EdgeConv presents a high computational load as it requires the evaluation of pairwise k-nearest neighbor queries.

### 2.3. Hybrid-Based Feature Encoders

Recent works attempting to combine the best of both grid- and point-set-based methods have shown the best results in 3D object detection. The authors of [9] proposed the PointPillar method, a hybrid approach that combines both voxelization and point-set-based approaches. In this method, the 3D space is first converted into 3D pillars, and then PointNet is applied to each pillar containing raw points for feature representation. The obtained feature representation is used by standard 2D convolutions for object detection. Although PointPillar is a computationally efficient approach, it relies heavily on manually adjusting the pillar size to improve its performance. The work in [35] proposed a two-stage feature framework, Fast Point R-CNN, where the initial feature representation from voxelization is fused with raw points to increase the accuracy of localization. Fast Point R-CNN achieved detection rate values of 15 FPS. Similarly, PointVoxel-RCNN (PV-RCNN) [36], an extension of the PointRCNN technique [32] and similar to spare-to-dense models [33], integrates two feature-encoding methods to aid in learning more-accurate 3D bounding boxes for 3D object detection. In PointVoxel-RCNN, the first stage encodes the scene from voxel to keypoint, and the second stage converts keypoints to grids for abstracting region-of-interest features.

## 3. Methodology

In this section, we formulate the problem of object detection in 3D point clouds. Then, we present our proposed 3D object detection pipeline. This pipeline includes an intensity-based encoder that enriches a voxel-wise set of geometric features. Finally, we describe our experimental setup, including the dataset used, preliminary exploration stages, evaluation strategy, and training process.

### 3.1. Problem Formulation

**Figure 1:**
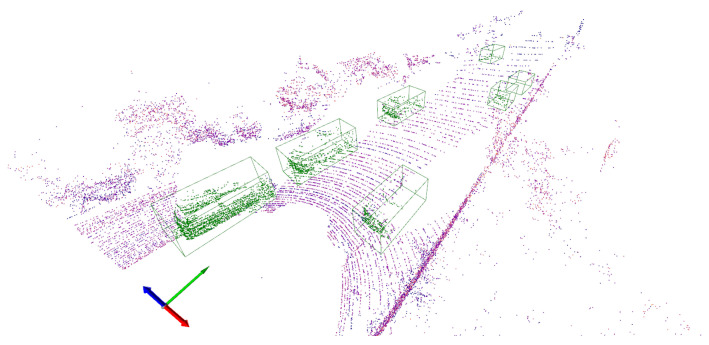
LiDAR-annotated scene consisting of a 3D point cloud and seven labelled objects indicated by green 3D bounding boxes.

Let us define a scene *S* as a 3D cloud point instance produced by a single sweep of a LiDAR sensor. In this paper, we will consider LiDAR sensors that record the 3D spatial coordinates $x_{i}$, $y_{i}$, and $z_{i}$ and the reflected intensity value $\rho_{i}$ of each scene point $p_{i}$. A scene is therefore a set of *N* points $S=\{p_{i}|1\leq i\leq N\}$, where $p_{i}={\left[x_{i},y_{i},z_{i},\rho_{i}\right]}^{T}$. In the context of 3D object detection, an annotated scene is defined as a scene *S* that is equipped with a label *Y* that includes the location, orientation, and class of every object in *S*. The location and orientation of an object can be described using a 3D bounding box, and examples of object classes in a typical urban scene include car and pedestrian classes. Figure 1 shows an example of an urban annotated scene consisting of a 3D point cloud produced by a LiDAR sensor.

Given a scene *S* produced by a LiDAR sensor, the 3D object detection problem consists of identifying the location, orientation, and class of all the objects in the scene. A possible solution to this problem comes in the form of a computational pipeline *l* that uses a scene *S* as an input and produces a predicted label $\hat{Y}$ as its output. Mathematically, we can express the functionality of this pipeline as follows:

(1) $$\hat{Y}=l\left(S\right)$$

To determine the class that one bounding box in $\hat{Y}$ belongs to, the computational pipeline *l* produces a probability value *c* for each class; this value can be interpreted as the pipeline’s confidence that the bounding box embeds an object of each defined class. If the confidence for a particular class is greater than a predetermined threshold $c_{T}$, the pipeline decides that an object of that class has been detected.

**Figure 2:**
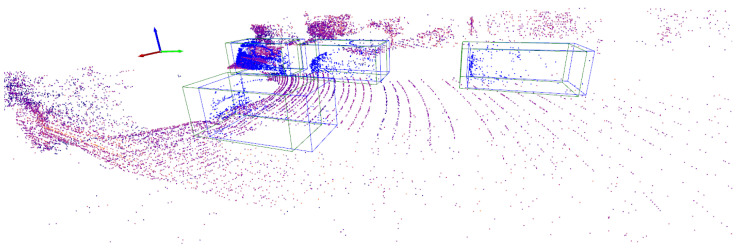
Bounding boxes corresponding to true objects (green) and objects predicted by a detection pipeline (blue). True and predicted objects are matched by computing the degree of overlap between their bounding boxes.

The performance of 3D object detection pipelines can be evaluated using datasets of annotated scenes. If there is only one class of objects in a scene, the performance of a pipeline *l* can be assessed as follows. Firstly, given an annotated scene $S_{k}$ appended with a ground truth label $Y_{k}$ and a predicted label ${\hat{Y}}_{k}$ produced by pipeline *l*, objects in $\hat{Y}$ are matched with objects in *Y*. Matching is conducted by obtaining the Intersection over Union (IoU) between the bounding boxes of each pair of objects from $\hat{Y}$ and *Y*, respectively. The IoU, which we denote as *O*, is computed from the bounding box *B* of an object in *Y* and the bounding box $\hat{B}$ of an object in $\hat{Y}$ as follows:

(2) $$O=\frac{vol(B\cap \hat{B})}{vol(B\cup \hat{B})}$$

where vol(·) computes the volume of its 3D argument, ∩ is the intersection between two 3D objects, and ∪ is their union. IoU values close to one indicate that there is a high overlap between bounding boxes *B* and $\hat{B}$, whereas IoU values close to zero indicate limited overlap. Object matching occurs when the IoU value is above a predefined threshold $O_{T}$. In addition, if several bounding boxes in $\hat{Y}$ overlap with the same ground bounding box in *Y*, the one with the highest IoU value is selected. This process, which is known as non-max suppression, is used to eliminate potential duplicate detections. Figure 2 shows the bounding boxes in a predicted label $\hat{Y}$ produced by a pipeline *l* for a subset of points of the scene in Figure 1. The bounding boxes of the objects in *Y* are also shown, demonstrating that the bounding box of every object in *Y* overlaps with the bounding box of one object in $\hat{Y}$. In one case, the bounding box of one of the objects in *Y* overlaps with the bounding boxes of two objects in $\hat{Y}$. If both predicted bounding boxes are matched to the true object, non-max suppression is triggered to select the predicted object with the highest IoU value.

**Figure 3:**
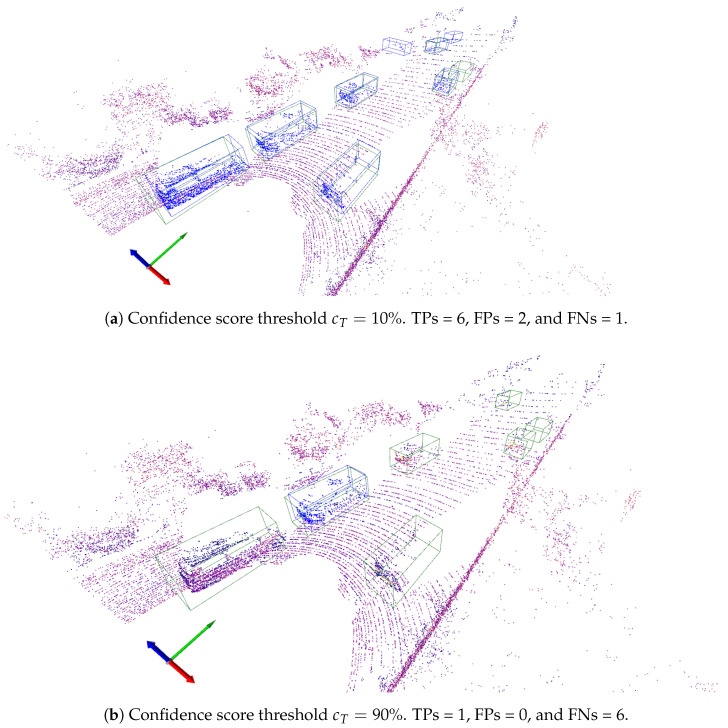
Superimposed in the annotated scene shown in Figure 1 are the bounding boxes of the objects detected by a pipeline *l* using a confidence score of 10% (**a**) and 90% (**b**). Ground truth objects are enclosed in a green bounding box, whereas predicted bounding boxes are blue. Predicted bounding boxes that match ground truth ones are TPs, whereas those that do not match a ground truth bounding box are FPs.

Once the matching process is completed, the concepts of true positive (TP), false positive (FP), and false negative (FN) are computed for every scene $S_{k}$. These concepts are defined as follows. An object in $\hat{Y}$ that matches one object in *Y* is a TP. In contrast, an object in $\hat{Y}$ that does not match any object in *Y* is a FP. Finally, an FN is any object in *Y* that is not matched by an object in $\hat{Y}$. It is worth noting that the values TP, FP, and FN depend on the confidence threshold $c_{T}$. In general, low $c_{T}$ values will result in many FPs and few FNs, and vice versa. This is illustrated in Figure 3a,b for the given scene instance, which shows, alongside the bounding boxes of the true objects, the bounding boxes of the objects predicted by a pipeline *l* using two different confidence thresholds, namely, $c_{T}=0.1$ and $c_{T}=0.9$. For $c_{T}=0.1$, the prediction pipeline produces 6 TPs, 2 FPs, and 1 FN. In contrast, using the confidence threshold $c_{T}=0.9$ leads to 1 TP, 0 FPs, and 6 FNs.

By aggregating the TP, FP, and FN values across all scenes $S_{k}$ in a dataset, we can compute the precision γ and recall *r* metrics of the pipeline *l* for a given confidence threshold $c_{T}$:

(3) $$\gamma =\frac{\sum_{k}{\mathrm{TP}}_{k}}{\sum_{k}{\mathrm{TP}}_{k}+\sum_{k}{\mathrm{FP}}_{k}}$$

(4) $$r=\frac{\sum_{k}{\mathrm{TP}}_{k}}{\sum_{k}{\mathrm{TP}}_{k}+\sum_{k}{\mathrm{FN}}_{k}}$$

where TP*_k_*, FP*_k_*, and TN*_k_* denote the TP, FP, and TN values for scene *k*. By gradually changing the confidence threshold from $c_{T}=0$ to $c_{T}=1$, a precision-recall curve can be obtained. The area under the precision–recall curve defines a performance metric known as the average precision (AP). The AP, which we denote as $\bar{\gamma }$, can be estimated from a dataset of annotated scenes:

(5) $$\bar{\gamma }=\frac{1}{L}\sum_{l}\max_{r\in R_{l}}\left[\gamma \left(r\right)\right]$$

where $R_{l}$ denotes the *l*-th segment resulting from partitioning the recall interval [0, 1] into *L* equal parts, and γ(r) is the precision of pipeline *l* when its corresponding recall value is *r*. Given a collection of 3D object detection pipelines, the AP value can be used as a metric to compare their performance.

**Figure 4:**
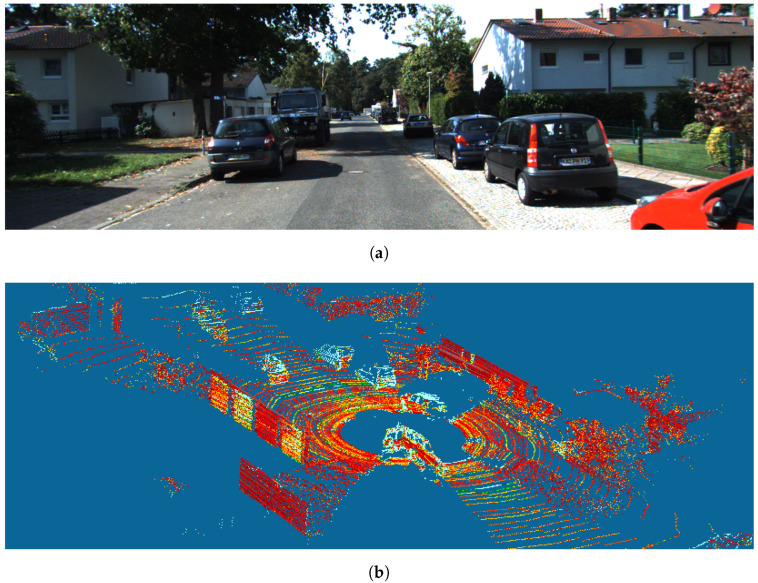

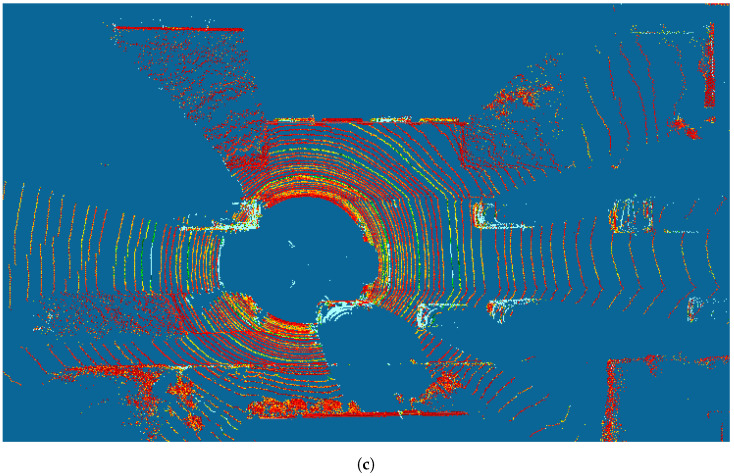
Example of a scene from the KITTI dataset: (**a**) 2D camera image, (**b**) 3D LiDAR point cloud, and (**c**) resulting 2D BEV point cloud. The BEV point cloud is produced by projecting the 3D LiDAR point cloud onto a top-down view plane. Objects recognizable in the 3D LiDAR point cloud (**b**) are also recognizable in the 2D BEV point cloud (**c**).

In the case of 3D scenes consisting of objects of multiple classes, we decompose the problem into several detection problems by considering each class separately. For instance, in a traffic scene consisting of cars, pedestrians, and bicycles, we would formulate three separate problems focusing on car detection, pedestrian detection, and cycle detection, respectively. Finally, BEV approaches provide an alternative to directly detecting objects in a 3D point cloud. By projecting 3D point clouds onto a 2D plane corresponding to a top-down view, BEV allows the problem of 3D detection to be recast as 2D detection. Figure 4 illustrates the principle of BEV. A 2D camera image and 3D LiDAR point cloud captured during the same urban traffic scene are shown in Figure 4a,b, respectively. The BEV point cloud resulting from projecting the 3D LiDAR point cloud onto a top-down view plane is shown in Figure 4c. Objects in the original 3D point cloud scene also appear in the BEV point cloud, which allows us to recast a 3D-object-detection-and-localization problem as a BEV 2D-object-detection-and-localization problem.

### 3.2. 3D Object Detection Pipeline

**Figure 5:**
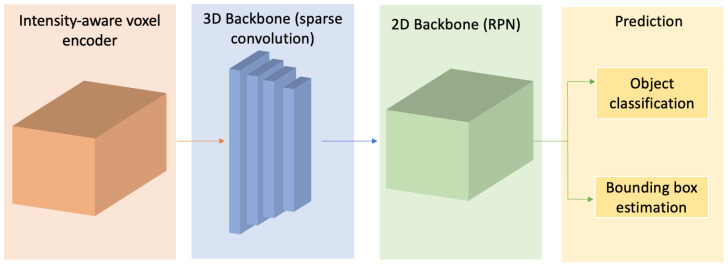
Our proposed 3D object detection pipeline consists of three stages, namely, an intensity-aware voxel encoder, which includes intensity features; a 3D backbone for dense feature extraction; and a 2D backbone that produces the final prediction (object classification and bounding box estimation).

Our proposed intensity-aware voxel encoder combines both geometric and intensity features of 3D point clouds and can be embedded within 3D object detection pipelines. To evaluate our intensity-aware voxel encoder, we embedded it within a single-stage 3D object detection pipeline. This single-stage 3D object detection pipeline is illustrated in Figure 5 and consists of a voxel-wise feature map generation stage, followed by a 3D backbone stage in which convolution operations are used to produce dense feature maps, and finally a 2D backbone stage for producing the final prediction, namely, object classification and 3D bounding box estimation. Within this pipeline, our proposed encoder generates voxel-wise feature maps by extracting geometric and intensity histogram features for each voxel in a parallel fashion. The three stages of the 3D object detection pipeline are described below.

#### 3.2.1. Intensity-Aware Voxel Feature Encoding

**Figure 6:**
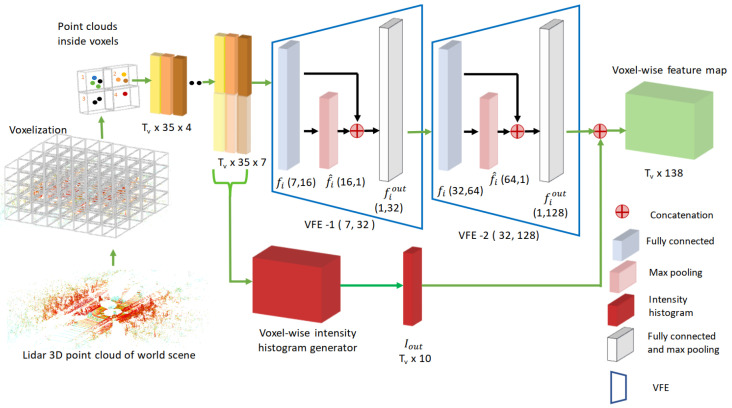
Architecture of the proposed intensity-aware voxel encoder. After voxelization, a scene is represented as a tensor with dimensions of $T_{v}\times 35\times 4$, where $T_{v}=T_{D}\times T_{H}\times T_{W}$, and $T_{D}$, $T_{H}$, and $T_{W}$ are the number of voxels along the depth, height, and width dimensions of the scene. After augmentation, a tensor whose dimensions are $T_{v}\times 35\times 7$ is generated and then processed via cascaded encoders VFE-1 and VFE-2. A voxel-wise intensity histogram $I_{out}$, whose dimensions are Tv×10, is concatenated to the output of VFE-2, whose dimensions are $T_{v}\times 128$, to produce the final $T_{v}\times 138$ voxel-wise feature map.

Our proposed voxel encoder is illustrated in Figure 6. The first step is scene voxelization. Given a 3D box with dimensions of D×H×W containing scene *S* and a predefined voxel with dimensions of $v_{D}\times v_{H}\times v_{W}$, we first partition the 3D box into a grid with the following dimensions: $T_{D}\times T_{H}\times T_{W}=\left(v_{D}/D\right)\times \left(v_{H}/H\right)\times \left(v_{W}/W\right)$. Once the voxel grid has been generated, points occupying each voxel are identified and grouped. Due to the sparse nature of 3D point clouds, some voxels have a large number of points, whereas others might have fewer points.

Two voxel-feature-encoding (VFE) stages convert the sparse 3D point cloud into a dense feature representation. VFE stages operate voxel-wise as follows. Let *V* be a collection of points $p_{i}$ within a given voxel. First, with an eye on computational efficiency, a subset of 35 points is randomly extracted from among all the points within each voxel. Given this random subset of points, an augmented representation ${\hat{p}}_{i}$ for each point $p_{i}$ is obtained by including the offset between each point and the mean of the voxel point cloud with the following coordinates: $\left(v_{x},v_{y},v_{z}\right)$. This augmented representation is defined as ${\hat{p}}_{i}={\left[x_{i},y_{i},z_{i},\rho_{i},x_{i}-v_{x},y_{i}-v_{y},z_{i}-v_{z}\right]}^{T}$. Each augmented point ${\hat{p}}_{i}$ is then transformed using a fully connected network into a complex feature $f_{i}$. The purpose of this network is to aggregate element-wise features, and it encodes the shape of the surface presented within a voxel. The fully connected network consists of a linear layer, batch normalization, and a rectified linear unit layer. After obtaining the element-wise feature representation $f_{i}$, we perform max pooling on $f_{i}$ to obtain a locally aggregated feature ${\hat{f}}_{i}$. Then, each complex feature $f_{i}$ is concatenated with ${\hat{f}}_{i}$ to form a point-wise concatenated feature $f_{i}^{out}$. Our intensity-aware voxel feature encoder includes two VFE blocks, namely, VFE-1 followed by VFE-2. The output from VFE-2 is concatenated with an intensity vector $I_{out}$ produced by an intensity histogram generator that operates in a voxel-wise fashion. In our study, the intensity histogram generator uses 10 bins and normalised intensity values within the range of 0 to 1.

#### 3.2.2. 3D and 2D Backbone Stages

A 3D backbone stage inspired by [31] was implemented. This backbone performs 3D sparse convolutions, which aggregate additional context with the feature descriptor produced by the intensity-aware voxel feature encoder. After performing the 3D sparse convolutions and reshaping the feature vector, a 2D backbone implementing the RPN is used for classification and 3D bounding box estimation. The RPN has two output heads, namely, a classification head and a regression head. The classification head is used to predict an object’s class, and the regression head is used to produce an estimation of the object’s bounding box. To improve the computational efficiency of the RPN stage, we use a set of predefined bounding boxes called anchors, each of which is associated with a different object class. Specifically, in a scenario where we are interested in detecting objects from three classes, e.g., ’Car’, ’Pedestrian’, and ’Cyclist’, three anchor boxes are created.

The detailed architectures of both 3D and 2D backbones follow the architectures presented in [31,36]. The 3D backbone stage uses sparse convolutions for dimensionality reduction and feature extraction. Convolutions are followed by batch normalization and rectifier linear unit activation. Sparse convolutional layers use a kernel size of (3, 1, 1) and a stride of (2, 1, 1) and produce a feature map with 128 output channels. This feature map is then processed via the 2D backbone stage, which consists of two blocks, each having five layers of 2D convolutions. Using the notation $Conv2D(C_{out},k,s,p)$ to describe a 2D convolutional layer, where $C_{out}$ represents the number of output channels, *k* is the kernel size, *s* stands for the stride, and *p* denotes the padding, in the first block, we employ Conv2D(128,3,1,1) layers, and in the second block, we employ Conv2D(256,3,1,1) layers. The final feature map produced by the 2D backbone is then sent to the classification and regression heads for object class prediction and bounding box estimation.

### 3.3. Experimental Setup

We used the KITTI dataset [37] to train and evaluate our proposed 3D object detection pipeline. Before training, we used the KITTI dataset and the Canadian Adverse Driving Condition (CADC) dataset [38] to explore the nature of the intensity value of LiDAR points in the context of object detection. The KITTI and CADC datasets, training environment, and evaluation approach are described below.

#### 3.3.1. Datasets

The KITTI dataset [37] is a popular dataset used for autonomous driving applications that offers annotated 2D camera images (375 × 1242 pixels) and 3D LiDAR images of 15K urban traffic scenes, together with other navigation data. Labels in the KITTI dataset include the location, size, and orientation of every object. Object location, size, and orientation are represented using a 3D bounding box in the LiDAR 3D image and a 2D bounding box in the corresponding 2D image. Object class name, truncation level, and occluded state are also given for each bounding box. The KITTI dataset defines nine different object classes, namely, ‘Car’, ‘Pedestrian’, ‘Cyclist’, ‘Van’, ‘Truck’, ‘Person (sitting)’, ‘Tram’, ‘Misc’, and ‘Don’t-Care’. The truncation value describes the fraction of objects lying outside the image boundary. Finally, the occlusion level, which takes on the values 0 through 3, describes the degree to which an object is occluded by other objects in a scene, where 0 indicates clearly visible and increasing values indicate greater occlusions.

The CADC dataset [38] provides a collection of scenes captured under adverse weather conditions. The dataset consists of 56K 2D camera images with a resolution of (1280 × 1024) pixels and 7K LiDAR instances. The CADC dataset includes 10 annotation classes, namely, ‘Car’, ‘Pedestrian’, ‘Truck’, ‘Bus’, ‘Garbage Container on Wheels’, ‘Traffic Guidance Object’, ‘Bicycle’, ‘Pedestrian With Object’, ‘Horse and Buggy’, and ‘Animal’. In this work, the CADC dataset is used to explore the impact of adverse weather conditions on LiDAR intensity distributions.

A preliminary exploration was carried out to investigate the possible impact of the surrounding environment, including scattering media such as rain, snow, and fog, on the LiDAR intensity values associated with traffic objects. We explored LiDAR scenes recorded in clear weather conditions from the KITTI dataset and scenes recorded in adverse weather conditions from the CADC dataset. Our preliminary exploration produced average profiles for the intensity distributions of objects from different classes. Intensity distributions were obtained using kernel density estimation (KDE), a non-parametric method that expresses a distribution as a linear combination of kernel functions centered around each dataset sample. In our implementation of KDE, we chose a Gaussian kernel. The bandwidth of Gaussian kernels is a parameter that needs to be set before applying KDE. We used Scott’s estimation method to select the value of the bandwidth. Scott’s method produces a bandwidth value that minimizes the mean integrated square error of the estimated distribution.

#### 3.3.2. Training and Evaluation

We used the KITTI benchmark dataset [23] for training and evaluation. This benchmark dataset consists of 7481 training instances and 7518 testing instances. We split the benchmark training dataset further into two subsets consisting of 3712 instances for training and 3769 instances for validation. Validation was conducted in accordance with the protocol described in [2,9,27,36]. We trained and evaluated our 3D object detection pipelines using an RTX 3080 10GB GPU and an AMD RYZEN 9 3900 CPU using a Pytorch-based *mmdetection3d* framework. The CUDA mixed-precision method was employed during training, allowing us to combine FP16 (16-bit, half-precision) and FP32 (32-bit, single-precision) floating-point formats to enhance computational speed. We trained our 3D object detection pipeline in an end-to-end fashion using the AdamW optimization algorithm, using a decaying learning rate of 0.01.

We chose the AP $\bar{\gamma }$ as our prediction performance metric. We obtained AP values separately for the object classes ’Car’, ’Pedestrian’, and ’Cyclist’, and within each class for 3D point cloud detection and BEV detection. We followed the KITTI benchmark evaluation criterion, which is based on the PASCAL criterion [37,39], for 3D point clouds and 2D BEV point clouds. According to this criterion, different classes use different IoU thresholds $O_{T}$ to produce a match between an object in *Y* and an object in $\hat{Y}$. Specifically, the threshold value $O_{T}$ is 0.7 for ’Car’ objects and 0.5 for both ’Pedestrian’ and ’Cyclist’ objects. In addition to the AP, we obtained the detection rate of each 3D object prediction pipeline, measured in frames per second (FPS). In our study, we report both the original detection rate values, as reported by the authors and on our hardware. Finally, three evaluation scenarios of different difficulty levels, namely, ’Easy’, ’Moderate’, and ’Hard’, were considered. Each difficulty level defines a set of constraints on the characteristics of the objects that are included, and they are defined in Table 1. For instance, at the ’Easy’ difficulty level, only objects that are fully visible, truncated up to 15%, and have a bounding box whose height is 40 pixels are included. We compared our 3D object detection pipeline against state-of-the-art models in terms of AP and prediction rate. We used the AP values reported by their authors and obtained new detection rate values using our hardware to ensure fairness.

## 4. Results

**Figure 7:**
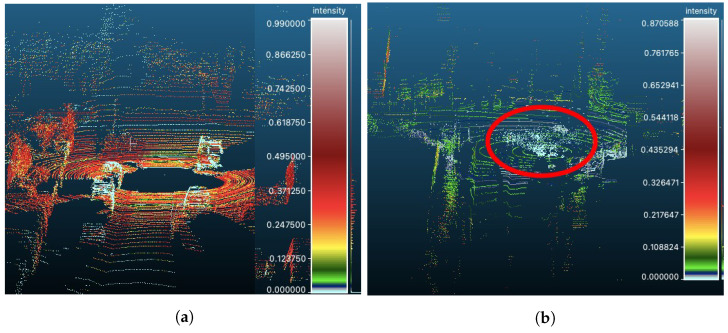
Examples of 3D LiDAR point cloud scenes where the reflected intensity $\rho_{i}$ has been-color coded. (**a**) KITTKI instance taken in clear weather conditions. (**b**) CADC instance taken during adverse weather conditions, where the highlighted region (red eclipse) shows low intensity values.

**Figure 8:**
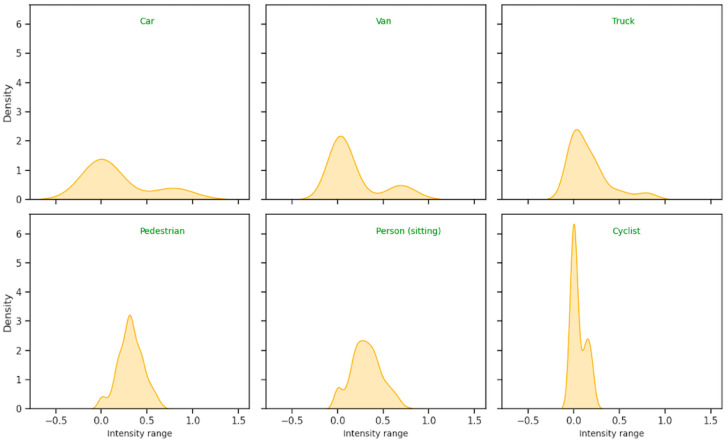
Intensity profiles of six objects defined in the KITTI dataset, obtained by averaging the intensity distributions obtained for each object by using KDE (‘Car’: 6647 objects; ‘Van’: 2106 objects; ‘Truck’: 1027 objects; ‘Pedestrian’: 1778 objects; ‘Person (sitting)’: 98 objects; ‘Cyclist’: 1132 objects). Intensity values below 0 or above 1 are artifacts due to the smoothing nature of the KDE method.

Figure 7 illustrates the effects of the weather conditions on the reflected intensities of LiDAR cloud points. Compared to clear weather conditions (Figure 7a), the spatial distribution of LiDAR points when there are adverse weather conditions (Figure 7b) is noisy due to collisions with air particles that do not correspond to true objects in a scene. In addition, the intensity values in adverse weather conditions are lower than those under clear weather conditions. LiDAR intensity values can therefore provide useful information about scenes that can contribute to the interpretation of LiDAR point spatial distribution. We also observed that different scene objects reveal different intensity profiles. The average intensity distributions for the objects ‘Car’, ‘Van’, ‘Truck’, ‘Pedestrian’, ‘Person (sitting)’, and ‘Cyclist’ in scenes from the KITTI dataset are shown in Figure 8. Each object reveals a unique intensity profile; consequently, the intensity distribution of the LiDAR points associated with an object can provide information about their class. We based the design of our proposed encoder on this fundamental observation, namely, that LiDAR intensity distributions depend on the class of the underlying object. Therefore, the LiDAR intensity distribution can be used to improve object classification.

**Figure 9:**
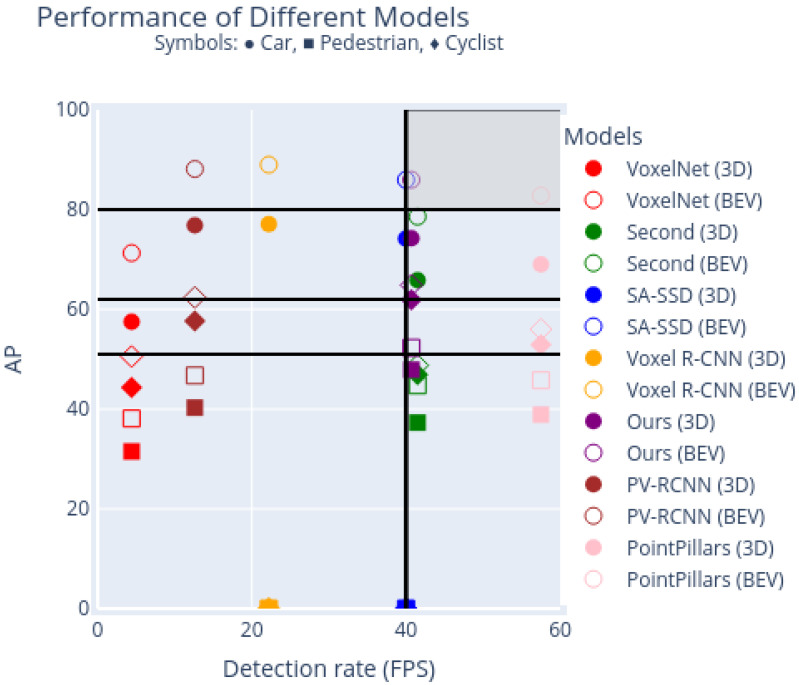
Visualisation in the AP × detection rate plane of the performance of the models shown in Table 6. AP values are obtained separately for ’Car’, ’Pedestrian’ and ’Cyclist’ objects, and for 3D and BEV detection modalities. Detection rates were computed using one single NVIDIA RTX 3080 GPU.

Table 2, Table 3 and Table 4 compare the AP values of the selected state-of-the-art 3D object detection models for ‘Car’, ‘Pedestrian’, and ‘Cyclist’ objects, respectively. The highest AP values in the ‘Hard’ evaluation scenario are highlighted, as they provide the strongest comparison between existing detection models. Despite its simplicity, the 3D object detection pipeline that we have designed to illustrate our proposed intensity-aware voxel encoder has a performance that is comparable with state-of-the-art models. Our proposed model ranks third in the AP benchmark for the ‘Car’ object, as shown in Table 2. Moreover, for the ’Pedestrian’ and ’Cyclist’ classes, our 3D object detection pipeline demonstrates superior performance, as shown in Table 3 and Table 4, respectively. Therefore, not only does this comparatively simpler architecture have a performance that is close to that of the more complex state-of-the-art models in regard to the ’Car’ benchmark, it also surpasses this performance for the ’Pedestrian’ and ’Cyclist’ benchmarks. Computational performance is compared in Table 5 and Table 6, which show the detection rates reported by the authors and the detection rates obtained in our computing environment, respectively. Our 3D object detection pipeline, which includes our proposed intensity-based encoder, achieved the second-highest detection rate, namely, 40.7, among the considered 3D object detection pipelines. It is worth noting that PointPillars, the model that achieved the highest frame rate, also presents a comparatively lower AP. The detection and computational performance of every 3D object detection pipeline under consideration are summarized in Figure 9, where the detection rate and AP for ‘Car’, ‘Pedestrian’, and ‘Cyclist’ objects under 3D detection and BEV detection are represented as coordinates on a 2D plane. The highest-performing models are situated close to the upper right corner, where AP is close to 100 and the detection rate is close to the real-time value of 60 FPS. Figure 9 demonstrates that the simple 3D object detection pipeline built around our intensity-aware voxel encoder achieves results that are superior to those obtainable by state-of-the-art models.

## 5. Conclusions and Discussion

In this paper, we have presented an intensity-aware voxel encoder for 3D LiDAR object detection and localization. The proposed encoder achieves AP values comparable to the state-of-the-art models while yielding higher detection rates during inference. In addition to this, a computationally efficient implementation of a voxel-wise histogram generator has been developed. Our results indicate that 3D object detection pipelines simpler than the state of the art can be developed to achieve accurate and robust 3D detection. The combination of our feature extractor and histogram generator can contribute to the development of 3D object detection models with higher inference rates.

Our preliminary analysis of the reflected intensity values of points associated with objects of different classes in the KITTI dataset suggests that each class of objects has a different intensity signature; therefore, LiDAR intensity values can be useful during 3D LiDAR object detection. In addition, we compared the intensity values from scenes under favorable and adverse weather conditions and observed the impact of rain and snow on the spatial distribution of LiDAR points and their intensity values. Based on this preliminary exploration, we proposed an intensity-aware voxel encoder that generates intensity histograms of point clouds within each voxel to capture the intensity profiles of objects within voxels. Voxel intensity histograms were then integrated as features together with conventional voxel feature sets.

We built a simple 3D object detection pipeline that included our intensity-aware voxel encoder to evaluate the potential impact of voxel intensity features on detection performance. Using the KITTI test dataset, we compared the detection performance and computational performance of our 3D object detection pipeline with state-of-the-art 3D object detection pipelines. Our 3D object detection pipeline outperforms the state-of-art models in regard to the ’Pedestrian’ and ’Cyclist’ classes. Although grid and point set-based feature encoders can implicitly consume intensity information together with the spatial distribution of 3D point clouds, our proposed encoder stands out in that it explicitly builds LiDAR intensity distributions by generating intensity histograms.

Current state-of-the-art 3D object detection pipelines have significantly improved over the past years with respect their performance on clear-weather datasets, but their performance deteriorates considerably when they are applied to adverse weather scenarios [15,43]. The reason for this deterioration is that LiDAR 3D point clouds are highly susceptible to adverse weather conditions or scattering media (fog, snow, rain, or dust) [17,18,19,20,21]. Therefore, adverse weather conditions produce noisy 3D point clouds that result in poor 3D object detection accuracy. To contribute to the development of 3D perception, academic and industrial partners have shared their pools of ever-growing datasets obtained under different environmental conditions while using different sensing modalities including LiDAR, cameras, and radar. Most of these benchmark datasets were recorded in clear weather [7,23,44], and some were obtained under adverse weather conditions [24,25,45]. We hypothesize that intensity-aware encoders might also improve 3D object detection performance under adverse weather conditions. Therefore, a future avenue for research on our proposed model will be to investigate its impact on 3D object detection performance under a wider range of weather conditions. This investigation should include adverse weather augmentation approaches to create controlled datasets of different complexity. Having robust 3D object detection pipelines that perform well under any weather conditions and in real-time is essential to achieve level 5 autonomy, which is defined as autonomy with no human intervention in any driving conditions. Due to the diversity of hazardous situations, collecting a complete dataset seems impractical. Employing simulation techniques based on physical and behavioral models of traffic objects and actors in different weather conditions is a promising direction. Creating custom simulated environments with complex situations can help develop robust and accurate 3D detection and tracking models for autonomous vehicles.

## Figures and Tables

**Table 1:** Evaluation difficulty levels.

Difficulty Level	Min. Bounding Box Height	Max. Occlusion Level	Max. Truncation
Easy	40 Px	0 (Fully visible)	15%
Moderate	25 Px	1 (Partly occluded)	30%
Hard	25 Px	2 (Difficult to see)	50%

**Table 2:** Performance comparison of 3D object detection pipelines applied to the KITTI test set for the ‘Car’ object. “L” indicates LiDAR-only method, while “R + L” indicates multi-modality method including both RGB images and LiDAR sensors.

Method	Modality	3D Detection (Car)			BEV Detection (Car)		
		Easy	Medium	Hard	Easy	Medium	Hard
MV3D [27]	R + L	74.97	63.63	54.00	86.62	78.93	69.80
AVOD-FPN [28]	R + L	83.07	71.76	65.73	90.99	84.82	79.62
F-PointNet [40]	R + L	82.19	69.79	60.59	91.17	84.67	74.77
UberATG-MMF	R + L	88.40	77.43	70.22	93.67	88.21	81.99
SECOND [31]	L	83.34	72.55	65.82	89.39	83.77	78.59
PointPillars [9]	L	82.58	74.31	68.99	90.07	86.56	82.81
PointRCNN [36]	L	86.96	75.64	70.70	92.13	87.39	82.72
STD [33]	L	87.95	79.71	75.09	94.74	89.19	86.42
Part-$A^{2}$-Net [14]	L	85.94	77.86	72.00	89.52	84.76	81.47
PV-RCNN [36]	L	90.25	81.43	76.82	94.98	90.65	86.14
Voxel R-CNN [2]	L	90.90	81.62	**77.06**	95.52	91.25	**88.99**
Ours	L	88.88	79.27	74.27	92.97	88.70	85.97

**Table 3:** Performance comparison of 3D object detection pipelines on the KITTI test set for the ‘Pedestrian’ object. “L” indicates LiDAR-only method, while “R + L” indicates a multi-modality method including both RGB images and LiDAR sensors.

Method	Modality	3D Detection (Pedestrian)			BEV Detection (Pedestrian)		
		Easy	Medium	Hard	Easy	Medium	Hard
AVOD-FPN [28]	R + L	50.46	42.27	39.04	58.49	50.32	46.98
F-PointNet [40]	R + L	50.53	41.15	38.08	57.13	49.57	45.48
SECOND [31]	L	51.45	41.92	38.89	58.69	50.13	46.84
PointPillars [9]	L	51.85	41.58	39.37	58.77	50.35	46.13
PointRCNN [36]	L	53.29	43.47	38.35	60.02	48.72	44.55
STD [33]	L	54.49	44.50	42.36	59.72	51.12	48.04
Part-$A^{2}$-Net [14]	L	53.42	43.29	40.29	59.86	50.57	46.74
PV-RCNN [36]	L	53.77	43.59	40.29	59.80	50.57	46.74
Voxel R-CNN [2]	L	54.95	44.52	41.25	60.74	50.58	46.74
Ours	L	61.62	53.74	**47.90**	65.75	58.76	**52.30**

**Table 4:** Performance comparison of 3D object detection pipelines on the KITTI test set for the ‘Cyclist’ object. “L” indicates LiDAR-only method, while “R + L” indicates a multi-modality method including both RGB images and LiDAR sensors.

Method	Modality	3D Detection (Cyclist)			BEV Detection (Cyclist)		
		Easy	Medium	Hard	Easy	Medium	Hard
AVOD-FPN [28]	R + L	63.76	50.55	44.93	69.39	57.12	51.09
F-PointNet [40]	R + L	72.27	56.12	49.01	77.26	61.37	53.78
SECOND [31]	L	71.33	52.08	45.83	76.5	56.05	49.45
PointPillars [9]	L	77.1	58.65	51.92	79.9	62.73	55.58
PointRCNN [36]	L	74.96	58.82	52.53	82.56	67.24	60.28
STD [33]	L	78.69	61.59	55.3	81.36	67.23	59.35
Part-$A^{2}$-Net [14]	L	78.58	62.73	57.74	81.91	68.12	61.92
PV-RCNN [36]	L	78.6	63.71	57.65	82.49	68.89	62.41
Ours	L	83.61	65.88	**61.94**	85.71	69.12	**64.85**

**Table 5:** Reported detection rates on the KITTI benchmark, using IoU thresholds of 70, 50, and 50 for ‘Car’, ‘Pedestrian’, and ‘Cyclist’ objects, respectively.

Encoding Schemes	Models	Modality	Hardware	FPS
Grid-Based	VoxelNet [12]	L	Titan X	4.4
	MVX-Ne [30]	L	-	-
	Second [31]	L	GTX 1080 Ti	30.4
	SA-SSD [41]	L	GTX 1080 Ti	25.0
	Voxel R-CNN [2]	L	RTX 2080 Ti	25.2
	Ours	L	RTX 3080	40.7
Point-Based	Points [10]	L + R	GTX1080	1.3
	MV3D [27]	L + R	Titan X	2.8
Hybrid	PointPillars [9]	L	GTX 1080 Ti	42.4
	PV-RCNN [36]	L	GTX1080	8.9
	MMF [42]	L + R	GTX1080	0.08

**Table 6:** Detection rates on the KITTI benchmark running on an RTX 3080 GPU, using IoU thresholds of 70, 50, and 50 for ‘Car’, ‘Pedestrian’, and ‘Cyclist’ objects, respectively.

Encoding Schemes	Models	FPS
Grid-Based	VoxelNet [12]	4.4
	Second [31]	41.5
	SA-SSD [41]	40.0
	Voxel R-CNN [2]	22.2
	Ours	40.7
Hybrid	PointPillars [9]	57.5
	PV-RCNN [36]	12.6

## Data Availability

Data are contained within the article.
